# Identification of candidate genes and chemicals associated with osteonecrosis of femoral head by multiomics studies and chemical-gene interaction analysis

**DOI:** 10.3389/fendo.2024.1419742

**Published:** 2024-08-26

**Authors:** Xueliang Lu, Xu Wang, Pengbo Wang, Yingkang Zhu, Jun Liu, Gang Liu, Ruiyu Liu

**Affiliations:** ^1^ Department of Orthopedics, Second Affiliated Hospital of Xi’an Jiaotong University, Xi’an, Shaanxi, China; ^2^ Department of Orthopedics, First Affiliated Hospital of Henan University of Science and Technology, Luoyang, Henan, China; ^3^ Department of Orthopedics, Xi’an Daxing Hospital, Xi’an, Shaanxi, China

**Keywords:** osteonecrosis of femoral head, cartilage, methylation, transcriptomics, proteomics, chemical-gene interaction

## Abstract

**Objectives:**

In-depth understanding of osteonecrosis of femoral head (ONFH) has revealed that degeneration of the hip cartilage plays a crucial role in ONFH progression. However, the underlying molecular mechanisms and susceptibility to environmental factors in hip cartilage that contribute to ONFH progression remain elusive.

**Methods:**

We conducted a multiomics study and chemical−gene interaction analysis of hip cartilage in ONFH. The differentially expressed genes (DEGs) involved in ONFH progression were identified in paired hip cartilage samples from 36 patients by combining genome-wide DNA methylation profiling, gene expression profiling, and quantitative proteomics. Gene functional enrichment and pathway analyses were performed via Gene Ontology (GO) and Kyoto Encyclopedia of Genes and Genomes (KEGG) enrichment analyses. Functional links between proteins were discovered through protein−protein interaction (PPI) networks. The ONFH-associated chemicals were identified by integrating the DEGs with the chemical−gene interaction sets in the Comparative Toxicogenomics Database (CTD). Finally, the DEGs, including MMP13 and CHI3L1, were validated via quantitative real-time PCR (qRT−PCR) and immunohistochemistry (IHC).

**Results:**

Twenty-two DEGs were identified across all three omics levels in ONFH cartilage, 16 of which were upregulated and six of which were downregulated. The collagen-containing extracellular matrix (ECM), ECM structural constituents, response to amino acids, the relaxin signaling pathway, and protein digestion and absorption were found to be primarily involved in cartilage degeneration in ONFH. Moreover, ten major ONFH-associated chemicals were identified, including, benzo(a)pyrene, valproic acid, and bisphenol A.

**Conclusion:**

Overall, our study identified several candidate genes, pathways, and chemicals associated with cartilage degeneration in ONFH, providing novel clues into the etiology and biological processes of ONFH progression.

## 1 Introduction

Osteonecrosis of femoral head (ONFH) is a severely disabling disease that usually affects young adults (1). As the pathogenesis of ONFH remains elusive, there is no authorized effective treatment for ONFH. Approximately 65% of ONFH patients ultimately need total hip replacement (2), resulting in heavy medical and economic burdens on these patients. However, the etiology and pathogenesis of hip cartilage degeneration in ONFH are currently unknown. Recent studies have demonstrated that hip cartilage degeneration plays a crucial role in ONFH development, with cartilage degeneration occurring early in ONFH (3). The destruction of hip cartilage increases hip instability, exacerbates femoral head collapse, and accelerates the development of ONFH (4). Thus, hip cartilage degeneration therapy may retard or even reverse the destruction of the hip joint caused by ONFH.

Recently, numerous studies have verified the impact of abnormal DNA methylation on the pathogenesis of bone and cartilage diseases (5, 6). Transcriptomics can reveal the mechanisms of ONFH progression at the genetic level (7). Proteomics is the study of the characterization of proteins at a large scale and can be applied to studies of cartilage pathophysiology (8). However, owing to the complexity and variability of molecules, individual omics analyses have difficulty providing a systematic and comprehensive understanding of complex biological processes (9). With their ability to study biological phenomena comprehensively, multiomics analyses can integrate the interactions of molecules at different layers and help bridge the gap from genotype to phenotype (10), improving the predictive accuracy of disease biological processes (11). Integrated proteomic and metabolomic analyses of synovial fluid have identified classical complement pathways and proinflammatory cytokines (*IL-6*, *IL-8*, and *IL-18*) as potential biomarkers of osteoarthritis (OA) and identified metabolically distinct OA subgroups (12). Multiomics exploration of therapeutic strategies for rheumatoid arthritis revealed that phellodendrine could effectively inhibit the activity of synovial cells and reduce the expression levels of inflammatory factors to alleviate joint inflammation and cartilage injury (13). Multiomics classification helps to distinguish the aggressiveness of cartilage tumors (14). Therefore, multiomics analyses of hip cartilage in ONFH comprehensively reveal the biological process of disease progression, which contributes to ONFH treatment and prevention.

The interactions between environmental factors and genes are involved in the development of chronic diseases by regulating physiological processes (15). However, to date, there are no epidemiologic reports of ONFH worldwide (16), and the environmental factors that predispose patients to ONFH are still unknown. The Comparative Toxicogenomics Database (CTD) is a publicly accessible database providing data about chemical−gene interactions (17). Therefore, integrating differentially expressed genes (DEGs) identified via multiomics analyses with chemical−gene interaction sets from the CTD will reveal the etiology and high-risk populations of ONFH patients.

In this study, we applied integrated multiomics (genome-wide DNA methylation profiling, gene expression profiling, and quantitative proteomics) in ONFH hip cartilage to obtain a comprehensive molecular portrait of cartilage degeneration. The ONFH-associated chemicals were subsequently identified by integrating the DEGs with the chemical−gene interaction sets in the CTD. Finally, the DEGs were validated via quantitative real-time PCR (qRT−PCR) and immunohistochemistry (IHC).

## 2 Methods

### 2.1 Patients and samples

**Figure 1:**
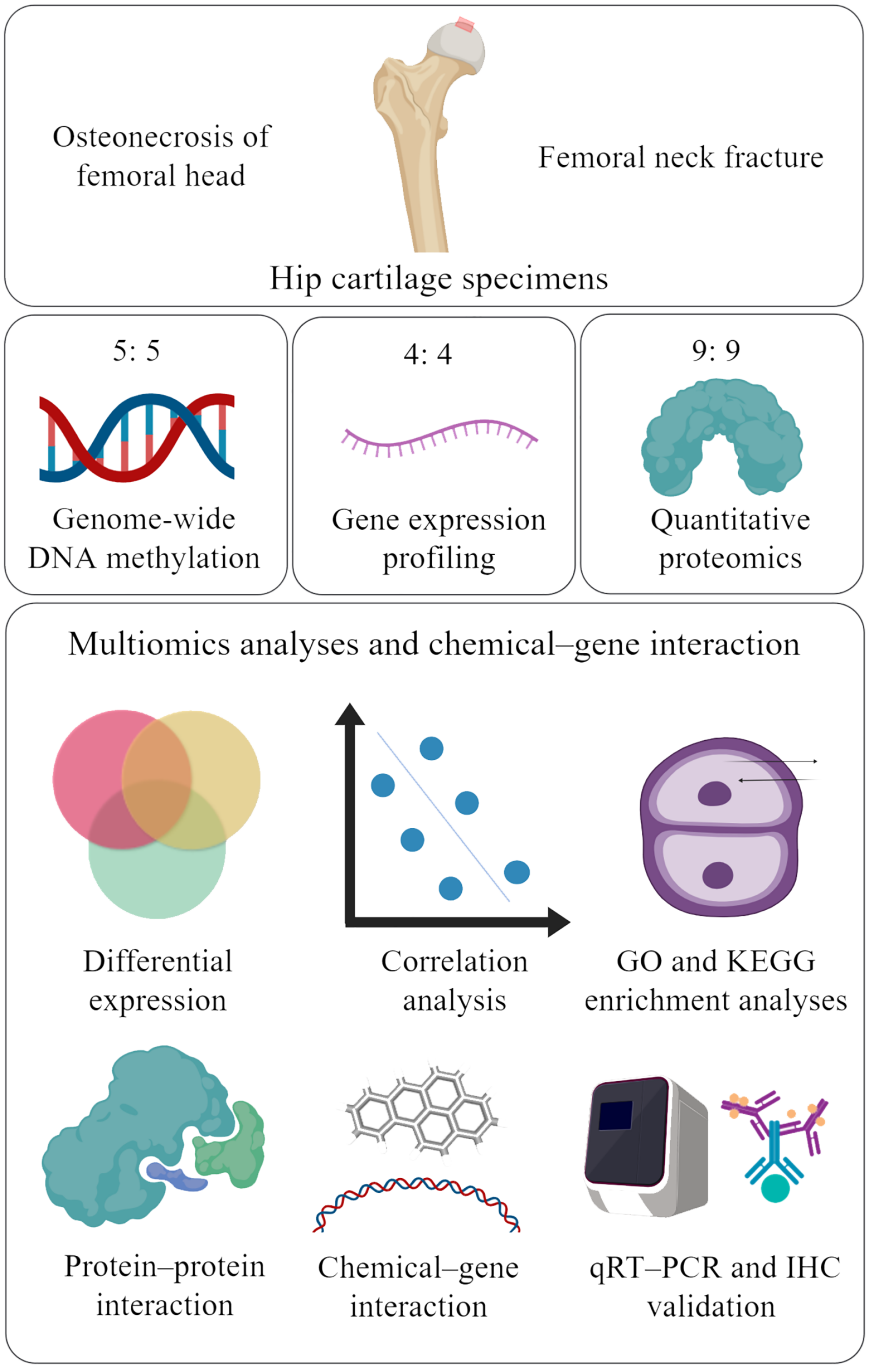
Study strategy (created with MedPeer). The red box shows the regions from which cartilage was collected from the femoral head. Cartilage samples from 18 ONFH patients and 18 femoral neck fracture controls were included in the study. Five, four, and nine ONFH control samples were selected for genome-wide DNA methylation profiling, gene expression profiling, and quantitative proteomics, respectively. The multiomics data were integrated to identify candidate genes, correlations, pathways, protein−protein interactions, and chemical−gene interactions. Finally, the differentially expressed proteins were validated via qRT−PCR and IHC. ONFH, osteonecrosis of femoral head; GO, Gene Ontology; KEGG, Kyoto Encyclopedia of Genes and Genomes; qRT−PCR, quantitative real-time PCR; IHC, immunohistochemistry.

A diagram of the research strategy, created with MedPeer (www.medpeer.cn), is shown in **Figure 1**. Briefly, human subjects were recruited from the Second Affiliated Hospital of Xi’an Jiaotong University. The clinical information of the study subjects is summarized in **Supplementary Table 1**. All the study subjects were Chinese Han individuals who were undergoing total hip replacement surgery. ONFH patients and control subjects were diagnosed according to clinical manifestations and radiologic imaging of the hip by two blinded ONFH experts. ONFH cartilage samples were harvested from patients with Ficat grade III ONFH (18). All cartilage samples were collected from anterosuperior portions of the femoral head (where the cartilage had collapsed in ONFH patients) within 2 h of total hip replacement. This study was approved by the Human Ethics Committees of Xi’an Jiaotong University. Informed consent documents were signed by all participants before they participated in this study. All methods were carried out in accordance with the relevant guidelines and regulations.

Ten hip articular cartilage samples were used for genome-wide DNA methylation profiling. Eight hip articular cartilage samples were used for gene expression profiling. Eighteen hip articular cartilage samples were subjected to quantitative proteomics analysis. ONFH samples from the above groups were paired with control samples.

### 2.2 Genome-wide DNA methylation

Genome-wide DNA methylation profiling was conducted via the Illumina Infinium HumanMethylation 850 BeadChip according to the manufacturer’s standard procedure. The percentage of methylated cytosine at a given CpG site was calculated as a β value, ranging from 0.0 (completely unmethylated) to 1.0 (completely methylated). A generalized linear mixed model was used to adjust for age and sex as covariates. We excluded CpG probes that mapped to sex chromosomes, cross-reacted with sex chromosomes or contained genetic variants. A total of 844,832 CpG sites that passed the quality control procedure were analyzed in this study. The Pearson correlation coefficients of the β values were calculated to evaluate the correlations of the samples.

Differentially methylated CpG sites were identified via the empirical Bayes moderated *t* test of the limma package (version 3.1.2) of R via DNA methylation M values. The Benjamini–Hochberg method was used to obtain an adjusted P value for each CpG locus. CpG sites with adjusted P values < 0.05 and |β value difference|>1 were considered significantly differentially methylated loci. For quality control, the CpG sites with missing values or P values > 0.05 in > 95% of the articular cartilage samples were eliminated.

### 2.3 Gene expression profiling

Total RNA was isolated from the cartilage samples via an Agilent Total RNA Isolation Mini Kit (Agilent Technologies, Santa Clara, CA, USA) following the manufacturer’s instructions. The isolated total RNA and complementary RNA (cRNA) contents were determined with an Agilent ND-1000 (Agilent Technologies). One microgram of labeled cRNA was mixed with hybridization buffer and hybridized to the Agilent Human 4x44K Gene Expression Microarray (v2, Agilent Technologies). The microarray data have been deposited in the Gene Expression Omnibus database [GEO: GSE74089]. The genes presenting both fold changes > 1.0 and P values < 0.05 were considered significantly differentially expressed. The false discovery rate (FDR) values were calculated via the permutation-based analysis algorithm of significance analysis of microarrays (SAM) (19).

### 2.4 Quantitative proteomics analysis

Isobaric tandem mass tag (TMT)-based quantitative proteomics analysis was applied to investigate the altered proteins associated with ONFH. LC−MS/MS analysis was performed using an Easy-nLC nanoflow HPLC system connected to a Q Exactive instrument (Thermo Fisher Scientific, MA, USA). The mass spectrometer was operated in positive ion mode. MS spectra were acquired over a range of 350–2000 m/z. The results were filtered on the basis of peptide and protein FDRs ≤ 1%. The relative peak intensities of the TMT reporter ions released in each of the MS/MS spectra were used. Only unique peptides obtained with a confidence percentage of > 95% were included. A fold change value of > 1 or < 1 and a P value of < 0.05 were used to identify upregulated or downregulated proteins.

### 2.5 Pathway and functional enrichment analyses

Pathway and functional enrichment analyses of DEGs from the integration of at least two omics methods were performed via Gene Ontology (GO) annotation (20) and Kyoto Encyclopedia of Genes and Genomes (KEGG) pathway mapping (21). Genes annotated to the same term were treated as “pathways”. The enrichment analyses of DEGs according to up- or downregulation were also conducted. Empirical p values for the enrichments were obtained from randomizations.

### 2.6 Protein−protein interaction network construction

The internet tool STRING (https://string-db.org) was used to analyze the probable interactions of differential proteins across all three omics levels (22). Interactions with summed scores greater than 0.15 were extracted to construct the PPI network. The PPI network was then visualized and subjected to further evaluation via the Cytoscape program (www.cytoscape.org). The Cytoscape MCODE plugin was adopted to identify crucial modules in the PPI network. The pathways involved in the crucial modules are shown in combination with KEGG.

### 2.7 Chemical−gene expression interactions

The CTD (http://ctdbase.org/) is a publicly available online database that provides access to data on chemical−gene interactions, chemical−disease associations, chemical−pathway associations, and chemical−phenotype associations. In the present study, we uploaded the differentially expressed genes across all three omics levels into the “Batch Query” of the CTD, selected the output of chemical−gene interactions, and screened the *H. sapiens* tissue data to obtain the top ten chemicals associated with ONFH on the basis of the number of chemical-acting genes.

### 2.8 qRT−PCR validation

The expression levels of mRNAs (MMP13 and CHI3L1) identified via transcriptomic data were validated via qRT−PCR in a cohort comprising four ONFH patients and four control samples. Total RNA from cartilage and subchondral bone tissue was extracted with TRIzol Reagent (Life Technologies, Carlsbad, CA) according to the manufacturer’s protocol. Then, 1 μg of total RNA was reverse transcribed via the Evo M-MLV RT Kit (Agbio, China), and qRT−PCR was performed via a QuantGene 9600 Real-Time PCR System (Bioer Technology, China). SYBR Green-based three-step qRT−PCR was performed via a SYBR Green Pro Taq HS HS qPCR Kit (Agbio). The primer sequences were retrieved from the online PrimerBank database (https://pga.mgh.harvard.edu/primerbank/index.html). GAPDH was used as the internal reference gene. Detailed information about the primers used for qRT−PCR is summarized in **Supplementary Table 2**.

### 2.9 IHC verification

The expression of MMP13 and CHI3L1 proteins in cartilage samples from ONFH patients and controls was verified via IHC. Briefly, cartilage tissue was fixed with paraformaldehyde, decalcified, and embedded in paraffin. The paraffin-embedded tissue sections were immersed in xylene to remove the paraffin, hydrated with a series of ethanol solutions, exposed to a 3% hydrogen peroxide solution for 10 minutes to deparaffinize and hydrate the cartilage sections, and then rinsed with PBS. After being blocked with QuickBlock™ Buffer (Beyotime Biotechnology, China) for 1 h, the sections were incubated with an MMP13 antibody (dilution: 1:100, ProteinTech Group; Chicago, IL, USA, no. 18165-1-AP) and a CHI3L1 antibody (dilution: 1:200, ProteinTech Group; Chicago, IL, USA, no. 12036-1-AP) at 4°C overnight. Following rinsing with PBS, the sections were exposed to an alkaline phosphatase-labeled secondary antibody (ZHONGSHAN Golden Bridge Biotechnology, Beijing, China) for 15 minutes at 37°C. They were then treated with streptavidin-horseradish peroxidase for another 15 minutes at the same temperature. Finally, the sections were stained with 3,3’-diaminobenzidine (DAB). The percentage of positive areas (cells) in the field of interest was calculated in the superficial, middle, and deep zones. Finally, differences in the expression of MMP13 and CHI3L1 proteins in the cartilage of the three ONFH patients and the three normal controls were analyzed via two-tailed Student’s t tests. The participants received information about the study, and their written consent was obtained.

## 3 Results

**Figure 2:**
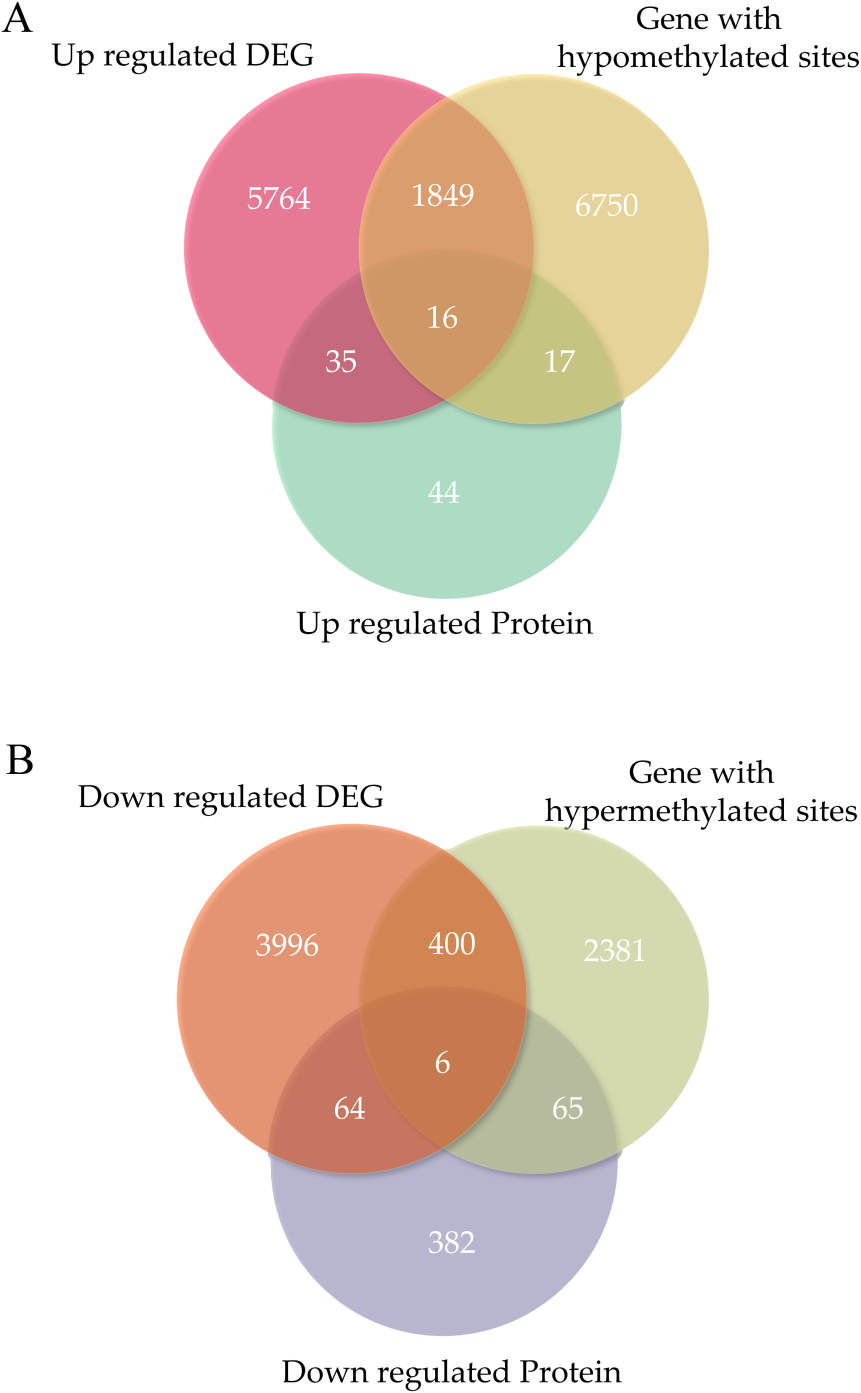
The overlapping genes were identified in each omics experiment. **(A)** Venn diagrams identifying the number of overlapping genes in each omics experiment for upregulation. **(B)** Venn diagrams identifying the number of overlapping genes in each -omics experiment for downregulation. DEGs, differentially expressed genes.

The abundance of 629 proteins was found to vary: 112 were observed at higher abundance and 517 at lower abundance in ONFH cartilage (**Figure 2**).

The expression of 12130 genes was significantly altered: 7664 genes were upregulated, and 4466 were downregulated in ONFH cartilage (**Figure 2**). The *MMP13* was significantly upregulated in the ONFH cartilage than in the control cartilage.

The expression of 11484 methylated sites significantly changed: 8632 genes exhibited hypomethylated sites and 2852 exhibited hypermethylated sites in ONFH cartilage (**Figure 2**).

### 3.1 Integration across multiple omics levels: methylation, RNA sequencing, and proteomics

At the three-omics level, 22 DEGs (including 16 upregulated genes and 6 downregulated genes) were found to be involved in the degenerative process of ONFH cartilage (**Supplementary Tables 3**, **4**). Among these, alterations in the expression of *MMP13*, *MMP2*, *COL3A1*, *COL5A2*, and *CHI3L1* have been previously reported in both ONFH and cartilage. In addition, 17 DEGs have not been reported in studies of ONFH; furthermore, seven genes have not been reported in cartilage. *MMP13*, *ECM1*, and *TPPP3*, which are upregulated in ONFH cartilage, were the three genes associated with the greatest log-fold changes in protein expression across the three omics methods. *CHI3L1*, *GDF10*, and *GNPDA1*, which are downregulated in ONFH cartilage, were the top three genes in terms of log-fold changes in protein expression across the three omics methods. The differentially expressed genes identified in this study that have never been previously mentioned in ONFH are summarized in **Supplementary Table 5**.

### 3.2 Proteomics and RNA sequencing

**Figure 3:**
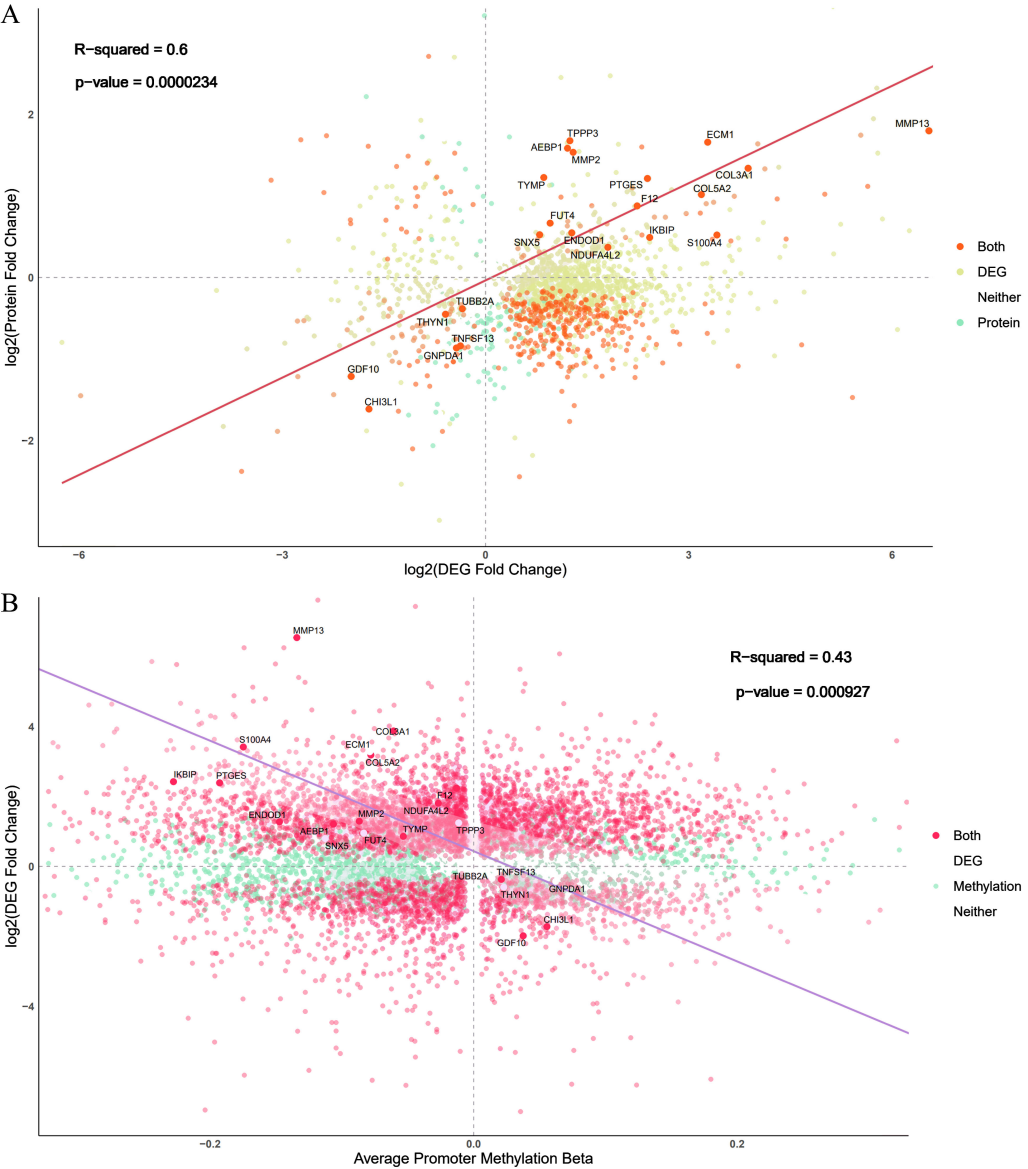
Comparison of changes identified in the omics experiments. **(A)** Comparison of the log-fold changes in both the proteomics and RNA-seq experiments. Each gene is represented as a single point, and the color corresponds to whether the gene is identified as differentially expressed via RNA-seq, proteomics analyses, or both. The screening threshold for the transcriptome and proteome was a P value less than 0.05. **(B)** Comparison of RNA-seq log-fold-change and average promoter region methylation changes in the differentially expressed genes. The screening threshold for methylation was a P value less than 0.05.

Among the proteins with evidence of differential abundance, 121 genes were also differentially expressed between the ONFH and control samples at the RNA level, of which 51 were upregulated and 70 were downregulated at both the RNA and protein levels in ONFH cartilage. Considering the RNA and protein changes in ONFH cartilage samples, we identified a significant positive correlation (**Figure 3A**).

### 3.3 Methylation and RNA sequencing

Among the 8632 hypomethylated sites, 1865 genes were also upregulated at the RNA level in ONFH cartilage. Among the 2852 hypermethylated sites, 406 genes were also downregulated at the RNA level in ONFH cartilage. A direct comparison of promoter region methylation and gene expression revealed the expected negative correlation between promoter region methylation and gene expression (**Figure 3B**).

### 3.4 Proteomics and methylation

Among these upregulated proteins, 33 hypomethylated sites were identified at the methylation level. Moreover, 71 hypermethylated sites were identified in the downregulated proteins at the methylation level.

### 3.5 Enrichment analyses involved in ONFH progression

**Figure 4:**
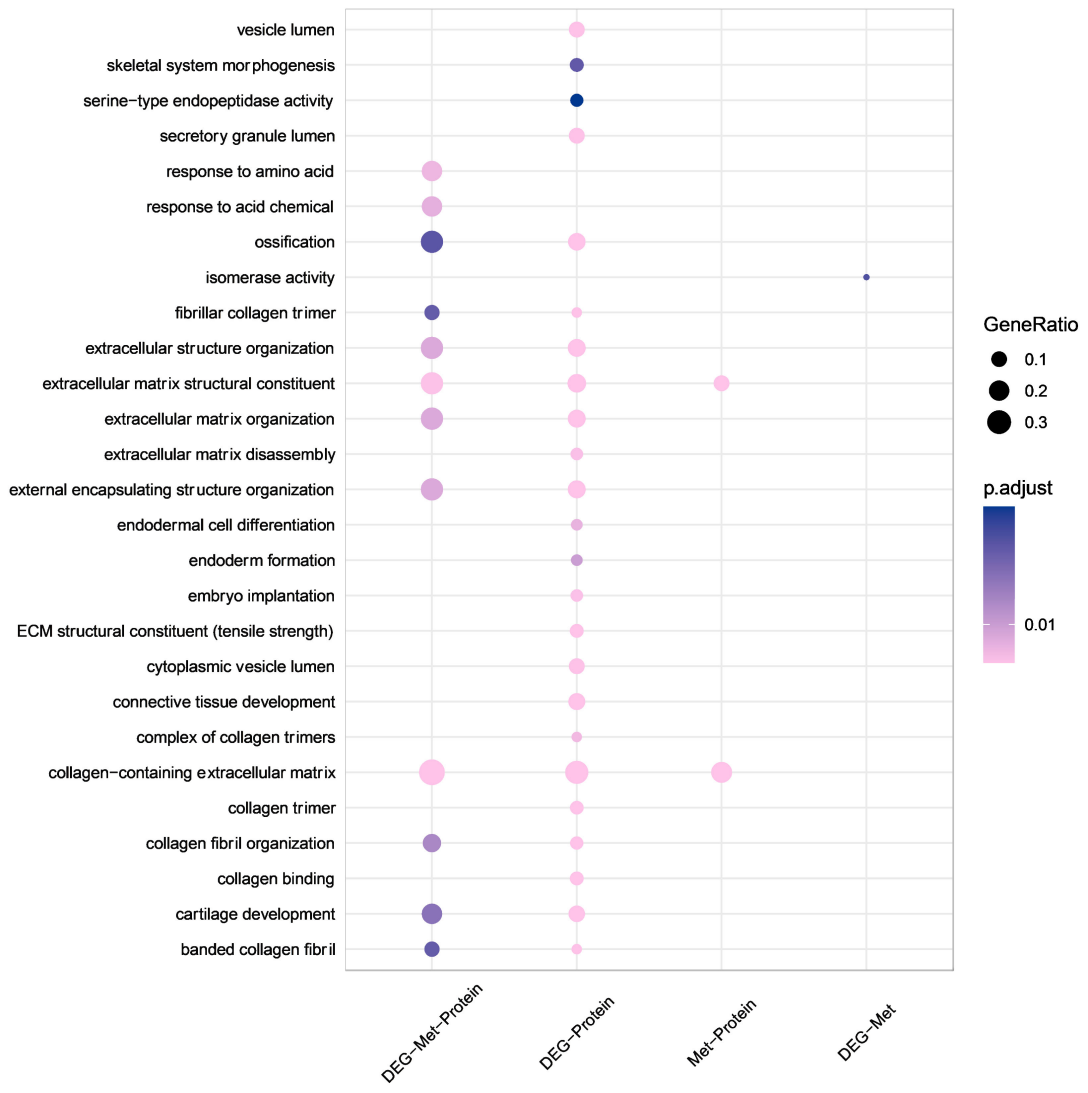
GO enrichment analysis at multiple omics levels. Larger bubble size represents a greater number of genes involved. The shapes are colored from pink to purple, representing low to high p.adjust. GO, Gene Ontology.

According to the results of the GO enrichment analysis (**Figure 4**), the DEGs at the three multiomics levels were primarily involved in the biological processes of the collagen-containing extracellular matrix, the extracellular matrix structural constituent, and the response to amino acids, which contributed to ONFH progression. Collagen-containing extracellular matrix, extracellular matrix structural constituent, external encapsulating structure organization, ossification, and cartilage development were enriched at the transcriptional and proteomic levels. Collagen-containing extracellular matrix and extracellular matrix structural constituents were also enriched at the methylation and proteomics levels. There was no common enriched pathway among the transcriptional and methylation levels. The detailed results of the GO enrichment analysis are shown in **Supplementary Figure 1**.

The results of the KEGG pathway analysis revealed that the relaxin and bladder cancer signaling pathways were enriched at three multiomics levels (**Supplementary Figure 1**). Amyotrophic lateral sclerosis, Huntington disease, Parkinson disease, and ribosomes were enriched at the transcriptional and methylation levels. Protein digestion and absorption, ECM-receptor interaction, and focal adhesion were enriched at the transcriptional and proteomic levels. Fatty acid degradation was enriched at the methylation and proteomics levels. The enrichment analysis results of the DEGs are shown in **Supplementary Figures 2**-**4**.

### 3.6 PPI network and KEGG pathway

**Figure 5:**
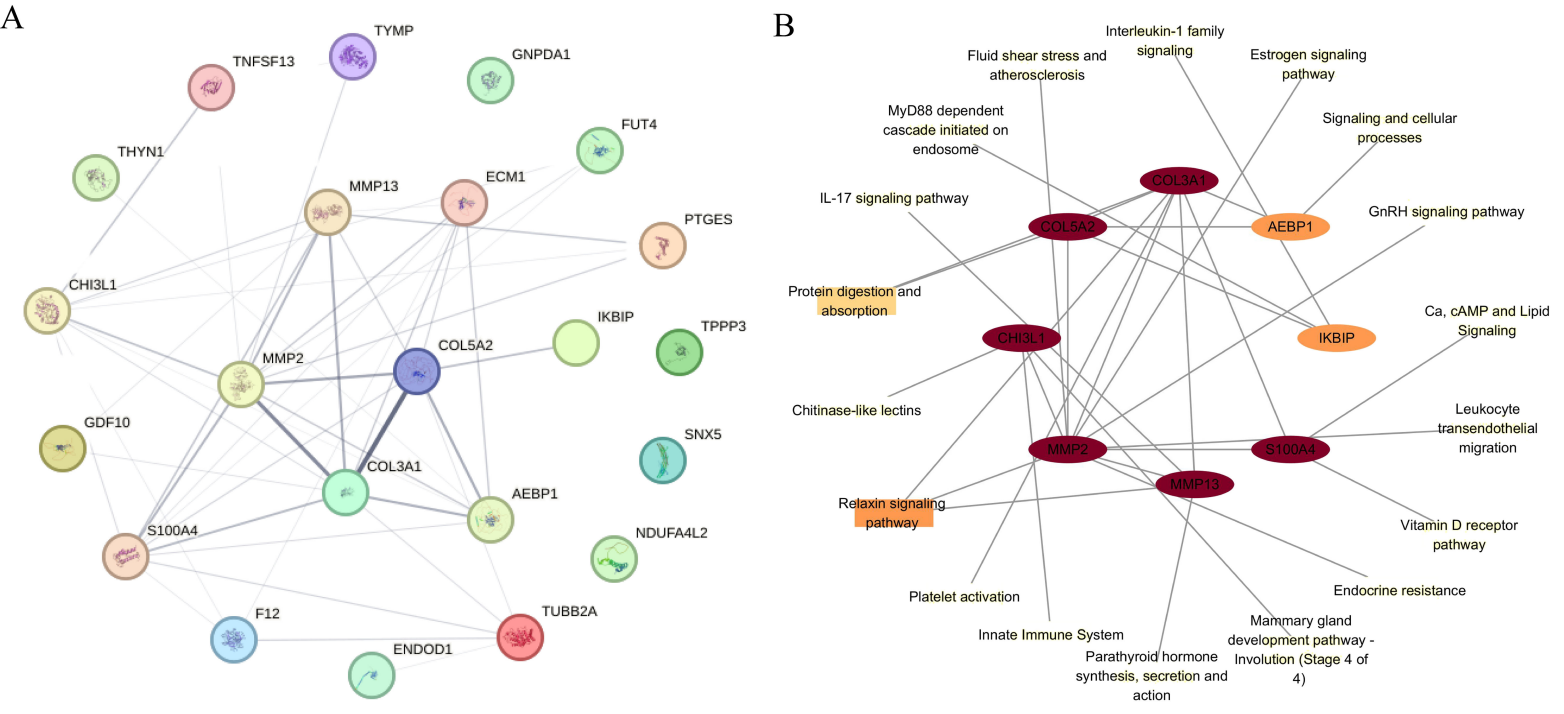
PPI analysis and KEGG pathway. **(A)** The PPI network of differentially regulated proteins across all three omics levels. **(B)** Eight proteins in ONFH cartilage were identified on the basis of their degree of interaction in the PPI network. The KEGG pathways associated with the eight proteins are indicated. The ellipse indicates proteins, and the rectangle indicates the KEGG pathway or biological process. The shapes are colored from yellow to red, representing low to high degrees. PPI, protein–protein interaction; KEGG, Kyoto Encyclopedia of Genes and Genomes.

The functional links between 22 differential proteins across all three omics levels were analyzed via a protein–protein interaction (PPI) network from the Search Tool for the Retrieval of Interacting Genes/Proteins (STRING) database (**Figure 5A**). Eight genes, seven upregulated and one downregulated, were identified in ONFH cartilage on the basis of the degree of interaction in the PPI network. The KEGG pathways associated with the eight genes are shown in **Figure 5B**. The relaxin signaling pathway and protein digestion and absorption are involved mainly in the pathogenesis of ONFH in cartilage. MMP13 is associated mainly with the IL-17 signaling pathway, relaxin signaling pathway, and parathyroid hormone synthesis, secretion, and action. CHI3L1 is associated mainly with chitinase-like lectins, the innate immune system, and the mammary gland development pathway (Stage 4 of 4).

### 3.7 Chemical−gene expression interactions in ONFH

Six hundred and seventy-six chemicals associated with DEGs across all three omics levels were identified. The top ten chemicals associated with ONFH were identified on the basis of the number of genes on which the chemicals act, in the order of benzo(a)pyrene (BaP), valproic acid, bisphenol A (BPA), tobacco smoke contamination, cisplatin, estradiol, dexamethasone (Dex), abrine, doxorubicin, and cyclosporine (**Table 1**).

**Table 1:** The top ten significantly ONFH-associated chemicals identified through the CTD database.

Chemical name	MeSH® ID	Number of intervening genes
Benzo(a)pyrene	D001564	19
Valproic acid	D014635	18
Bisphenol A	C006780	17
Tobacco smoke pollution	D014028	17
Cisplatin	D002945	16
Estradiol	D004958	15
Dexamethasone	D003907	14
Abrine	C496492	13
Doxorubicin	D004317	13
Cyclosporine	D016572	12

### 3.8 qRT−PCR validation of mRNAs

**Figure 6:**
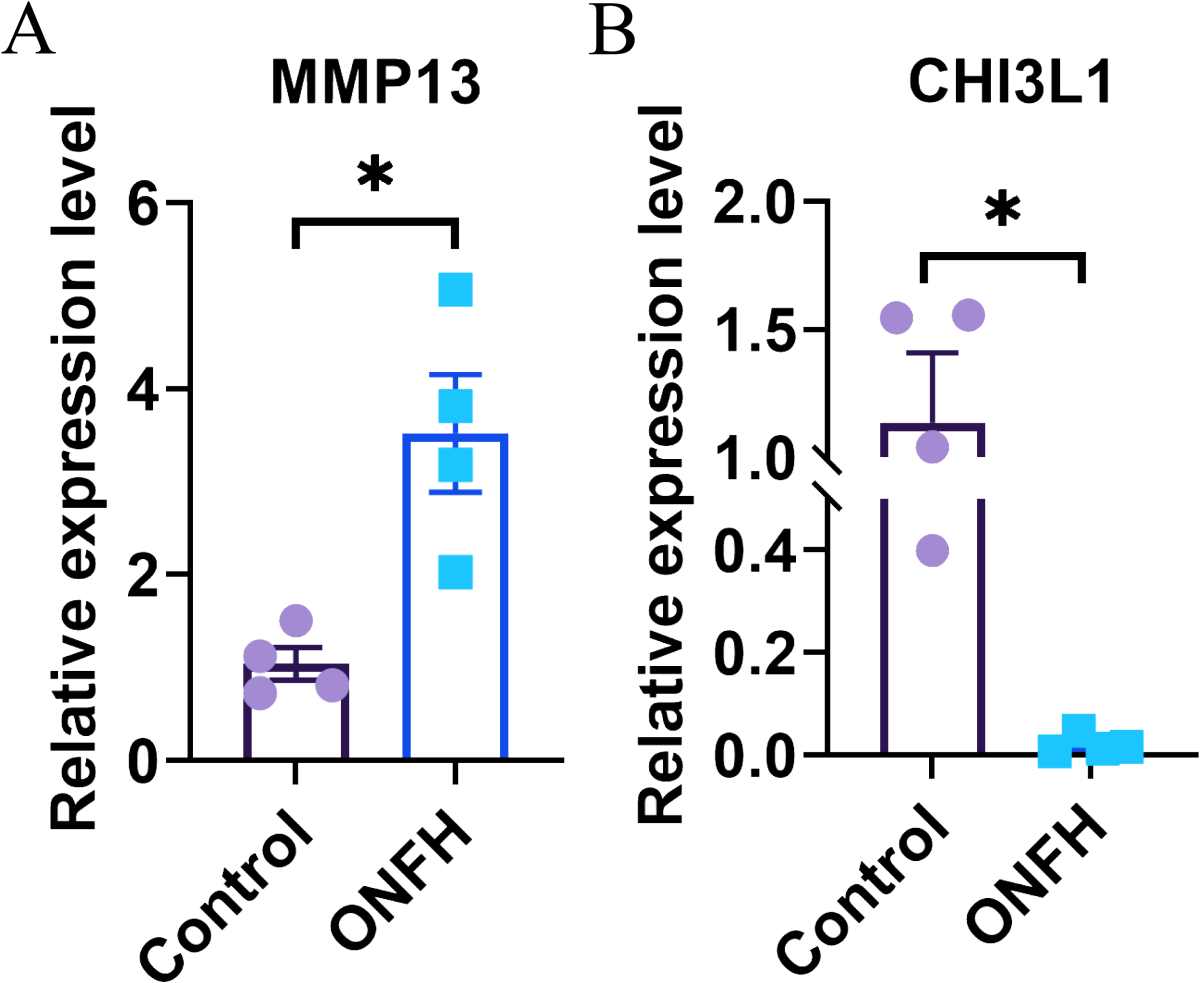
Results of qRT−PCR. **(A, B)** Confirmation of the transcriptomic results for mRNAs through qRT−PCR, with statistical significance at P < 0.05 (*). qRT−PCR, quantitative real-time PCR; ONFH, osteonecrosis of femoral head.

To validate the reliability of the transcriptomic data, we quantified *MMP13* and *CHI3L1* expression in four ONFH patients and four control patients via qRT−PCR. The results of qRT−PCR showed that the expression levels of *MMP13* and *CHI3L1* were consistent with those of the transcriptomic analysis (**Figure 6**).

### 3.9 IHC verification

**Figure 7:**
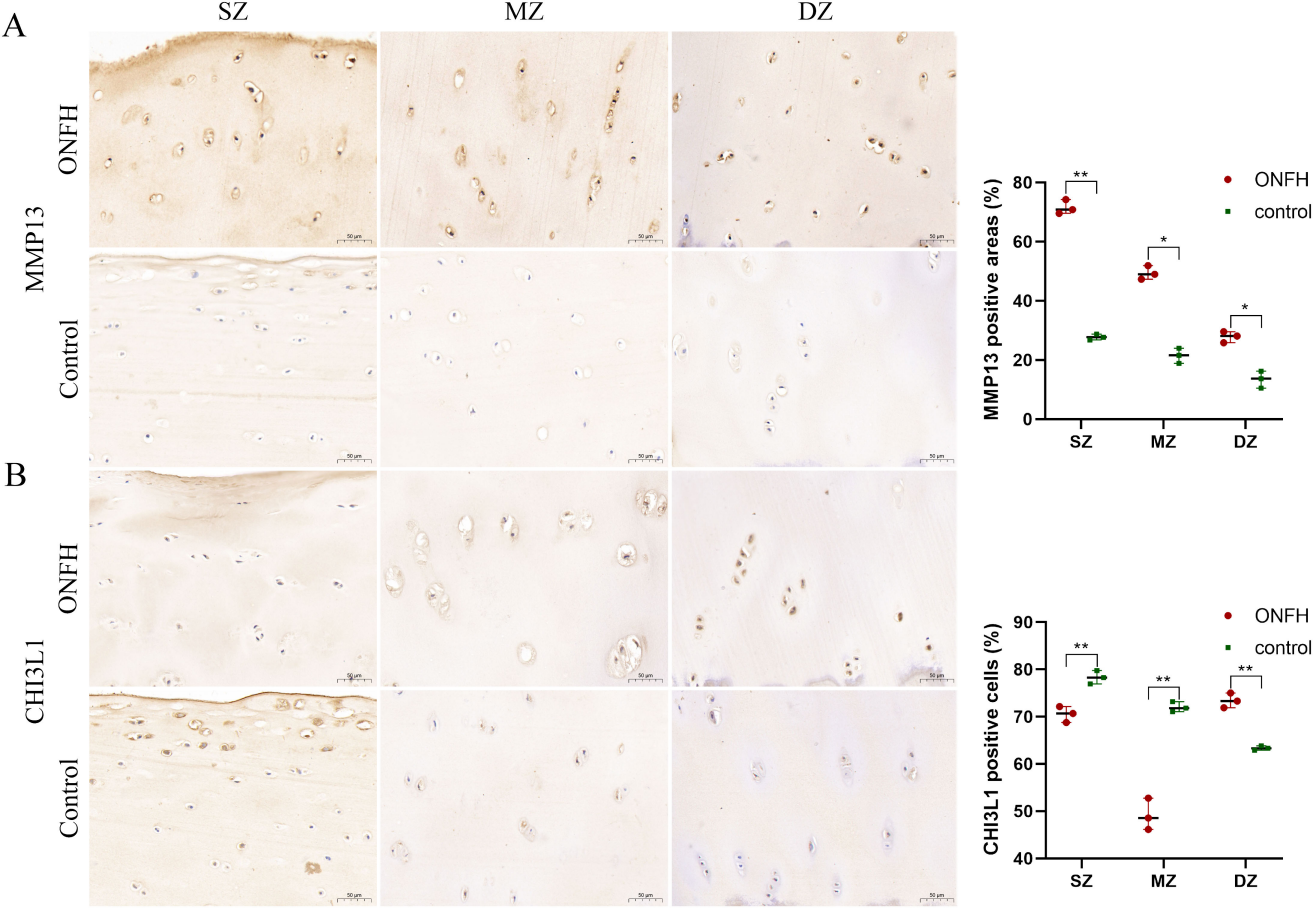
IHC results for the MMP13 **(A)** and CHI3L1 **(B)** proteins in hip cartilage from ONFH patients and fracture controls. Original magnification x300 of the SZ, MZ, and DZ. *P value < 0.05, **P value < 0.01. IHC, Immunohistochemistry; ONFH, osteonecrosis of femoral head; SZ, superficial zone; MZ, middle zone; DZ, deep zone. Scale bar: 50 μm.

IHC was used to evaluate MMP13 and CHI3L1 protein expression in ONFH and control tissues (**Figure 7**). The protein expression levels of MMP13 in the superficial zone (SZ), middle zone (MZ), and deep zone (DZ) of ONFH cartilage were significantly greater than those in the control cartilage (all P values were less than 0.05). The protein expression levels of CHI3L1 in the SZ and MZ of ONFH cartilage were significantly lower than those in the control cartilage (all P values were less than 0.01). However, the protein expression levels of CHI3L1 in the DZ of ONFH cartilage were significantly greater than those in the control (P values were less than 0.01). The immunohistochemical results were consistent with the results of the multiomics analysis.

## 4 Discussion

The pathophysiology of ONFH remains unclear. Recently, it has been increasingly recognized that cartilage degeneration plays a crucial role in ONFH progression (23). Previous studies of ONFH have focused mainly on single omics methods, such as methylation (24), transcription (25), or protein expression (26), or have combined up to two of these omics assays (27). However, single-omics analysis does not provide a systematic understanding of complex biological processes. This study provides comprehensive multiomics analyses of biological changes in ONFH cartilage across genome-wide DNA methylation profiling, gene expression profiling, and quantitative proteomics.

*MMP13* breaks down the ECM, efficiently cleaves type II collagen and participates in cartilage pathophysiologic processes. *MMP13* can degrade aggrecan, one of the most essential components of cartilage, and cause damage to cartilage (28). A previous study (28) confirmed increased levels of *MMP13* in patients with ONFH, which is consistent with our omics and IHC results. During the development of ONFH, the expression of chondrogenesis-related genes (*MMP13*) is significantly and progressively elevated (29). Previous studies have shown that *ECM1* binds to other extracellular matrix proteins, including collagen type IV, fibronectin, laminin 332, fibulin-1C/1D and MMP-9 (30). *ECM1* plays a role in endochondral bone formation (31), promotes the proliferation of endothelial cells, and induces angiogenesis (32). *TPPP3* enables the binding of tubulin, which is an early progenitor cell marker for both tendon and osteochondral cells. The heterotopic ossification-induced increase in *TPPP3*+ cells in a postoperative heterotopic ossification hip arthroplasty model led to ectopic cartilage formation (33). *COL3A1* encodes the pro-alpha1 chain of type III collagen, a fibrillar collagen found in extensible connective tissues. Type III collagen is also a vital regulator of the collagen fibrillar structure and biomechanics of articular cartilage (34). A study by Wang et al. indicated that *COL3A1* can be a biomarker for cartilage degradation in posttraumatic osteoarthritis (35). *S100A4* encodes a small calcium-binding protein that is commonly overexpressed in a range of different tumor types, and a growing body of evidence suggests that *S100A4* has an essential role in the process of cancer metastasis (36). The *S100A4* protein binds to receptors for advanced glycation end products and mediates the induction of *MMP13* in hip articular chondrocytes (37). Our findings indicate that *COL3A1* and *S100A4* are upregulated across multiple omics levels in ONFH cartilage and are significantly associated with ONFH cartilage degeneration. Moreover, additional studies should be conducted in the future.

*CHI3L1* is expressed and secreted by various cell types, including macrophages, chondrocytes, fibroblast-like synovial cells, and vascular smooth muscle cells (38). *CHI3L1* was localized in chondrocytes in the superficial and middle layers of the cartilage. *CHI3L1* reflects not only the degree of inflammation but also the metabolism of cartilage. Kawasaki et al. (39) reported that a high abundance of *CHI3L1* was correlated with Ficat stage III disease with collapsed femoral head. However, no other studies have reported a role for this gene in ONFH. We found that the protein expression of CHI3L1 was lower in chondrocytes and the superficial and middle layers of ONFH patients compared with controls. However, elevated expression in the deeper layers may occur because bone tissue necrosis first leads to inflammatory changes in the deeper layers of the cartilage. *GDF10* (also called *BMP-3b*), a member of the bone morphogenetic protein (BMP) family and the transforming growth factor-beta superfamily, has been reported to be an essential regulator of critical events in the processes of articular chondrocyte differentiation and bone formation (40, 41). However, the role of *GDF10* in ONFH is not fully understood. Our results revealed that *GDF10*, which is associated with cartilage degeneration, was downregulated at the omics level in ONFH patients. GNPDA1 is involved in the pathways of glycolysis and glycosaminoglycan metabolism. The downregulation of *GNPDA1* leads to a decreased energy supply, which is one of the mechanisms of cartilage degeneration in ONFH. *TNFSF13* (also called *APRIL*) is produced by myeloid cells and their precursors in the bone marrow (42) and is involved in the induction of the immunoglobulin switch and the survival of plasmocytes (43). It is involved in the development of systemic autoimmune diseases such as lupus erythematosus and rheumatoid arthritis. Moreover, it strongly promotes the proliferation of cancer cells and can be secreted by these cells (44). The downregulation of *TNFSF13* in ONFH cartilage may be the reason for corticosteroid-induced ONFH, and most patients with this condition experienced autoimmune diseases in the past and required long-term glucocorticoid treatment.

Homeostasis of the cartilage matrix is maintained by cartilage cells. The ECM, which is mainly composed of type II collagen and proteoglycans, is a significant component of cartilage and is essential for chondrocyte function. In addition to supporting chondrocyte growth, the ECM responds to external environmental stimuli through dynamic regulation to maintain articular cartilage homeostasis (45). The key enzymes involved in cartilage destruction are matrix-degrading enzymes, which include matrix metalloproteinases (*MMPs*) and disintegrin and metalloproteinase with thrombospondin-like motifs (*ADAMTSs*) (46). In current studies, aggrecan is mainly degraded by *MMPs* (*1*, *3* and *13*) and *ADAMTSs* (*4* and *5*), whereas collagen II is degraded by *MMPs*, mainly *MMP13 (* 47, 48). The results of this study revealed that in ONFH cartilage, *MMP13*, *MMP2*, and *ADAMTS5* are upregulated, which disrupts the homeostasis of the ECM and thus causes cartilage degeneration. Stabilizing the ECM in the future could delay the progression of ONFH. Amino sugars are important compounds required to form chondrocytes and represent one of the elementary units of the cartilage matrix and joint fluid (49). *CCN2* may regulate the levels of free amino acids in the extracellular matrix of cartilage under physiological conditions (50). A study revealed a lower amino acid content in OA articular and meniscal cartilage than in normal articular cartilage (51). The levels of amino sugars were reduced in the present study, which may also be a potential factor in ONFH cartilage degeneration.

Additionally, among the identified chemicals, Dex is a recognized exposure factor that contributes to ONFH and is commonly used to construct experimental models of ONFH (52). Treating traumatic joint injuries with Dex prevents the initial damage caused by the release of inflammatory cytokines and maintains the cartilage structure (53). However, repeated use of Dex at high doses over a long period of time may result in more harm than beneficial effects to the joint (54). Dex results in increased expression of *MMP13 (* 55) and *F12* mRNA (56). Long-term administration of Dex resulted in decreased subchondral bone mass and bone density in mice. Research has shown that Dex hardens the extracellular matrix of chondrocytes, partly by activating AKT, and accelerates the death of chondrocytes in the layer of hardened cartilage. In addition, Dex attenuates stress-responsive autophagy over time (57). Steroids produced through the OPG/RANK/RANKL signaling pathway can lead to an imbalance in the bone remodeling process of the femoral head, inhibiting bone formation and causing osteonecrosis (58). An association was found between the methylation of the *OPG*, *RANK* and *RANKL* genes and steroid-related ONFH (59). Tobacco smoke contamination results in increased expression of *CHI3L1* mRNA (60) and *GNPDA1* protein (61), leading to decreased expression of *MMP13 (* 62)*, S100A4 (* 63), and *FUT4* mRNAs (64). *CHI3L1* can be a potential marker for the severity and progression of osteoarthritis cartilage degeneration (65). *CHI3L1* enhances mitosis in chondrocytes through MAP kinase and PI3 kinase-mediated signaling and induces SOX9 and type II collagen synthesis (66). Smoking increases the risk of ONFH (67), which aligns with our findings. Estradiol not only has positive effects on bone growth and bone remodeling (68) but can also lead to adverse effects of venous thromboembolism (69). Altered DNA methylation has more recently been implicated in BaP-mediated toxicity. DNA methylation, gene mutations and DNA damage can influence alternative splicing during gene transcription (70). Selective splicing is a significant process that generates diverse proteins in more complex organisms (71). BaP causes abnormal selective splicing of specific genes (72). Analysis of the changes in gene expression revealed that various important pathways, such as drug metabolism signaling, NRF2-mediated oxidative stress responses, and glutathione-mediated detoxification, are affected by BaP. Furthermore, the activation of several disease pathways, including organism death, growth failure, and abnormal development of embryonic tissues, is predicted (73). NRF2 is a major regulator of intracellular antioxidants, and upregulation of its expression provides femoral protection in glucocorticoid-induced ONFH (74). BaP significantly suppressed early and late osteogenic differentiation and downregulated runt-related transcription factor 2, osteocalcin, and osteopontin during the induction of *BMP2* in mesenchymal stem cells (75). Moreover, BaP can impair the expression and function of the sonic hedgehog signaling pathway, perturbing the proliferation of chondrocytes and disturbing craniofacial skeletal development (76). Valproic acid decreases the expression of *MMP2* and *MMP9* mRNAs in osteosarcoma cells (77). Valproic acid results in increased methylation of the *FUT4* and *F12* genes (78). Previous studies have shown that valproic acid has a teratogenic effect on human chondrogenesis (79) and causes thrombocytopenia (80). Valproic acid counteracts the suppressive impact of glucocorticoids on the proliferation and osteogenesis of bone marrow−derived mesenchymal stem cells by suppressing apoptosis and enhancing the expression of proteins linked to osteogenesis. This mechanism may contribute to the prevention of glucocorticoid-induced ONFH in rats (81). BPA, a high-volume chemical with endocrine-disrupting properties, has been extensively studied in the context of bone metabolism. BPA results in decreased expression of *FUT4* mRNA (82). BPA inhibits osteoblast differentiation and bone formation by activating retinoic acid-related orphan receptor α (83), resulting in osteoporosis (84).

## 5 Conclusion

Our study identified 22 DEGs across all three omics levels and several crucial pathways in ONFH cartilage through multiomics analyses, which may help to elucidate the pathogenesis of cartilage degeneration in ONFH. Moreover, we identified ten major environmental factors associated with ONFH progression, which can aid the early prevention and treatment of ONFH.

## Data Availability

The datasets presented in this study can be found in online repositories. The names of the repository/repositories and accession number(s) can be found in the article/**Supplementary Material**.

## Supplementary material

Supplementary Figure 1 GO and KEGG enrichment analyses at the two- or three-omics level. GO, Gene Ontology; KEGG, Kyoto Encyclopedia of Genes and Genomes.

Supplementary Figure 2 Integrated enrichment analyses on the basis of adjustment direction at the two- or three-omics levels. **(A)** GO pathways of the upregulated genes. **(B)** KEGG pathway analysis of the upregulated genes. **(C)** GO pathways of the downregulated genes. **(D)** KEGG pathway analysis of the downregulated genes. The results of two- or three-omics enrichment analyses on the basis of reorientation. GO, Gene Ontology; KEGG, Kyoto Encyclopedia of Genes and Genomes.

Supplementary Figure 3 GO and KEGG enrichment analyses of the upregulated gene sets at the two- or three-omics levels. GO, Gene Ontology; KEGG, Kyoto Encyclopedia of Genes and Genomes.

Supplementary Figure 4 GO and KEGG enrichment analyses of the downregulated gene sets at the two- or three-omics levels. GO, Gene Ontology; KEGG, Kyoto Encyclopedia of Genes and Genomes.
