# Supplementary material for: Identification of candidate genes and chemicals associated with osteonecrosis of femoral head by multiomics studies and chemical-gene interaction analysis
Source: Front Endocrinol (Lausanne). 2024 Aug 26;15:1419742. doi: 10.3389/fendo.2024.1419742 (PMC11382631; doi:10.3389/fendo.2024.1419742)
Supplement: Supplementary Figure 1 — GO and KEGG enrichment analyses at the two- or three-omics level. GO, Gene Ontology; KEGG, Kyoto Encyclopedia of Genes and Genomes. [file DataSheet1.zip › Supplementary Table 1.docx]

**Supplementary Table 1**. Clinical data of the study subjects.

| Group | ONFH | | Fracture control | |
| --- | --- | --- | --- | --- |
|  | Age (years) | Sex | Age (years) | Sex |
| Genome-wide DNA methylation profiling | | | | |
| 1 | 50 | Female | 61 | Female |
| 2 | 54 | Male | 60 | Male |
| 3 | 52 | Male | 63 | Male |
| 4 | 51 | Male | 61 | Male |
| 5 | 55 | Male | 65 | Male |
| Gene expression profiling | | | | |
| 1 | 42 | Male | 45 | Male |
| 2 | 41 | Male | 42 | Male |
| 3 | 51 | Male | 53 | Male |
| 4 | 47 | Female | 47 | Female |
| Quantitative proteomics analysis | | | | |
| 1 | 56 | Male | 61 | Male |
| 2 | 51 | Male | 61 | Male |
| 3 | 52 | Female | 65 | Female |
| 4 | 46 | Male | 60 | Male |
| 5 | 55 | Male | 59 | Male |
| 6 | 50 | Female | 63 | Female |
| 7 | 50 | Male | 60 | Male |
| 8 | 45 | Male | 57 | Male |
| 9 | 48 | Female | 62 | Female |
| qRT‒PCR | | | | |
| 1 | 51 | Male | 57 | Male |
| 2 | 47 | Male | 47 | Male |
| 3 | 54 | Female | 46 | Female |
| 4 | 49 | Female | 52 | Female |
| IHC | | | | |
| 1 | 42 | Male | 46 | Male |
| 2 | 38 | Male | 41 | Male |
| 3 | 55 | Female | 53 | Female |
