# Supplementary material for: Identification of candidate genes and chemicals associated with osteonecrosis of femoral head by multiomics studies and chemical-gene interaction analysis
Source: Front Endocrinol (Lausanne). 2024 Aug 26;15:1419742. doi: 10.3389/fendo.2024.1419742 (PMC11382631; doi:10.3389/fendo.2024.1419742)
Supplement: Supplementary Figure 1 — GO and KEGG enrichment analyses at the two- or three-omics level. GO, Gene Ontology; KEGG, Kyoto Encyclopedia of Genes and Genomes. [file DataSheet1.zip › Supplementary Table 2.docx]

**Supplementary Table 2.** The qRT‒PCR primers used.

| **Gene Symbol** | **Primer sequence** | **Length** | **Amplicon Size** |
| --- | --- | --- | --- |
| MMP13 | F: 5′- CCAGACTTCACGATGGCATTG-3′ | 21 | 137 |
|  | R: 5′- GGCATCTCCTCCATAATTTGGC-3′ | 22 |  |
| CHI3L1 | F: 5′- GTGAAGGCGTCTCAAACAGG-3′ | 20 | 141 |
|  | R: 5′- GAAGCGGTCAAGGGCATCT-3′ | 19 |  |
| GAPDH | F: 5′-CTGGGCTACACTGAGCACC-3′ | 19 | 101 |
|  | R: 5′-AAGTGGTCGTTGAGGGCAATG-3′ | 21 |  |
