# Supplementary material for: Identification of candidate genes and chemicals associated with osteonecrosis of femoral head by multiomics studies and chemical-gene interaction analysis
Source: Front Endocrinol (Lausanne). 2024 Aug 26;15:1419742. doi: 10.3389/fendo.2024.1419742 (PMC11382631; doi:10.3389/fendo.2024.1419742)
Supplement: Supplementary Figure 1 — GO and KEGG enrichment analyses at the two- or three-omics level. GO, Gene Ontology; KEGG, Kyoto Encyclopedia of Genes and Genomes. [file DataSheet1.zip › Supplementary Table 5.docx]

**Supplementary Table 5.** The differentially expressed genes identified in this study that have never been previously mentioned in the context of ONFH.

| Gene | ONFH | Cartilage | Related pathways |
| --- | --- | --- | --- |
| FUT4 | Not | Mentioned | Blood group systems biosynthesis and Glycosaminoglycan metabolism |
| ECM1 | Not | Mentioned | Integrin Pathway and ERK Signaling |
| F12 | Not | Mentioned | Diseases of hemostasis and Agents Acting on the Renin-Angiotensin System Pathway |
| S100A4 | Not | Mentioned | Vitamin D receptor pathway and Ca, cAMP and Lipid Signaling |
| AEBP1 | Not | Mentioned | DNA-binding transcription factor activity and Calmodulin binding |
| PTGES | Not | Mentioned | Fatty acid metabolism and Metabolism |
| NDUFA4L2 | Not | Mentioned | Respiratory electron transport, ATP synthesis by chemiosmotic coupling, and heat production by uncoupling proteins |
| TPPP3 | Not | Mentioned | Tubulin binding |
| GDF10 | Not | Mentioned | Apoptotic Pathways in Synovial Fibroblasts and GPCR Pathway |
| TNFSF13 | Not | Mentioned | MIF Mediated Glucocorticoid Regulation and TNF Superfamily - Human Ligand-Receptor Interactions and their Associated Functions |
| ENDOD1 | Not | Not | Response to elevated platelet cytosolic Ca^2+^ |
| IKBIP | Not | Not | MyD88 dependent cascade initiated on endosome and Interleukin-1 family signaling |
| TYMP | Not | Not | Apoptotic Pathways in Synovial Fibroblasts and GPCR Pathway |
| SNX5 | Not | Not | Vesicle-mediated transport and trans-Golgi Network Vesicle Budding |
| GNPDA1 | Not | Not | Glycolysis (REACTOME) and Glycosaminoglycan metabolism |
| TUBB2A | Not | Not | Cooperation of Prefoldin and TriC/CCT in actin and tubulin folding and Golgi-to-ER retrograde transport |
| THYN1 | Not | Not | Induction of apoptosis |
