# Supplementary figures and images for: Identification of candidate genes and chemicals associated with osteonecrosis of femoral head by multiomics studies and chemical-gene interaction analysis
Source: Front Endocrinol (Lausanne). 2024 Aug 26;15:1419742. doi: 10.3389/fendo.2024.1419742 (PMC11382631; doi:10.3389/fendo.2024.1419742)

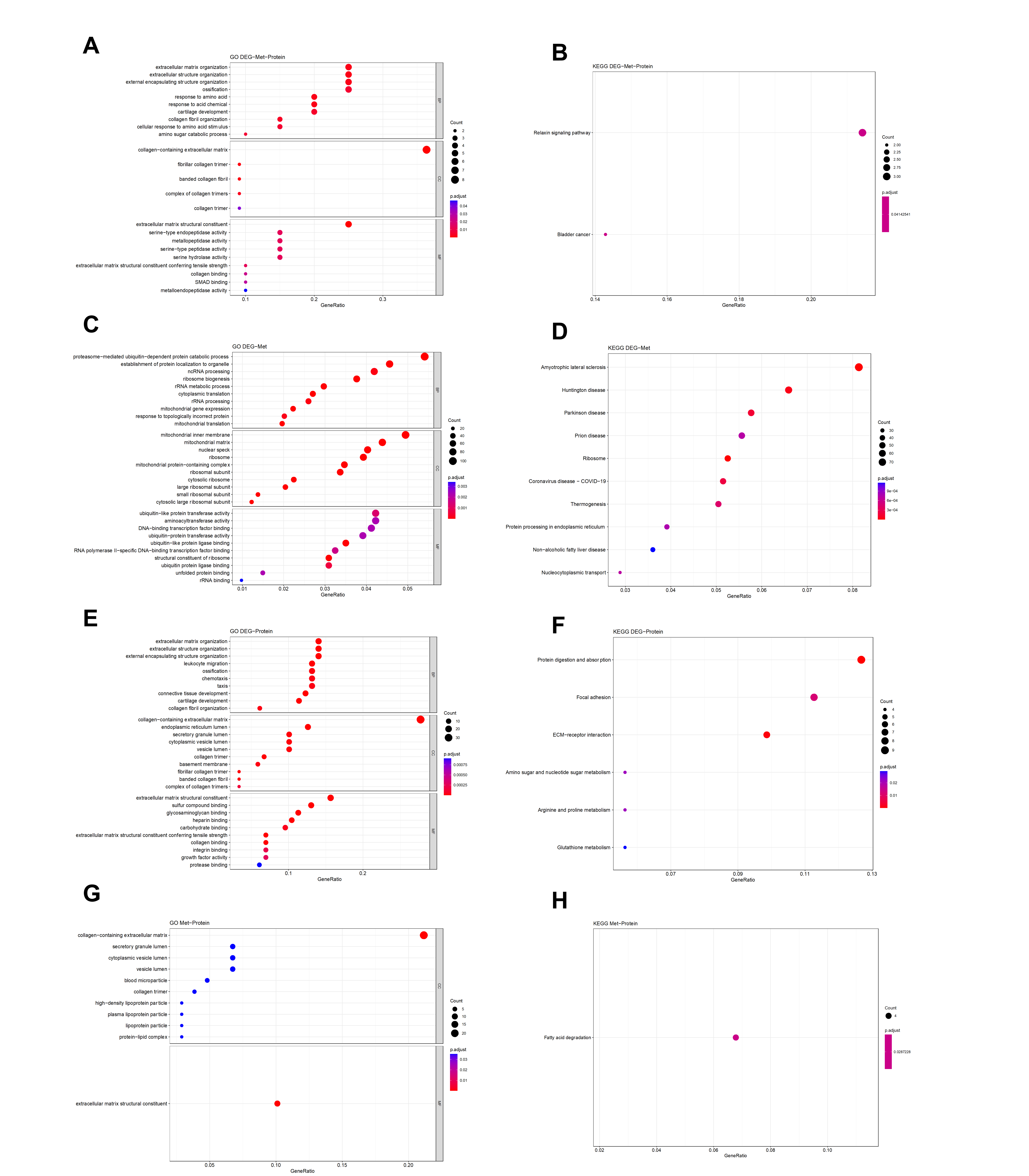

Supplement: Supplementary Figure 1 — GO and KEGG enrichment analyses at the two- or three-omics level. GO, Gene Ontology; KEGG, Kyoto Encyclopedia of Genes and Genomes. [file DataSheet1.zip › Supplementary Figure 1.TIF]

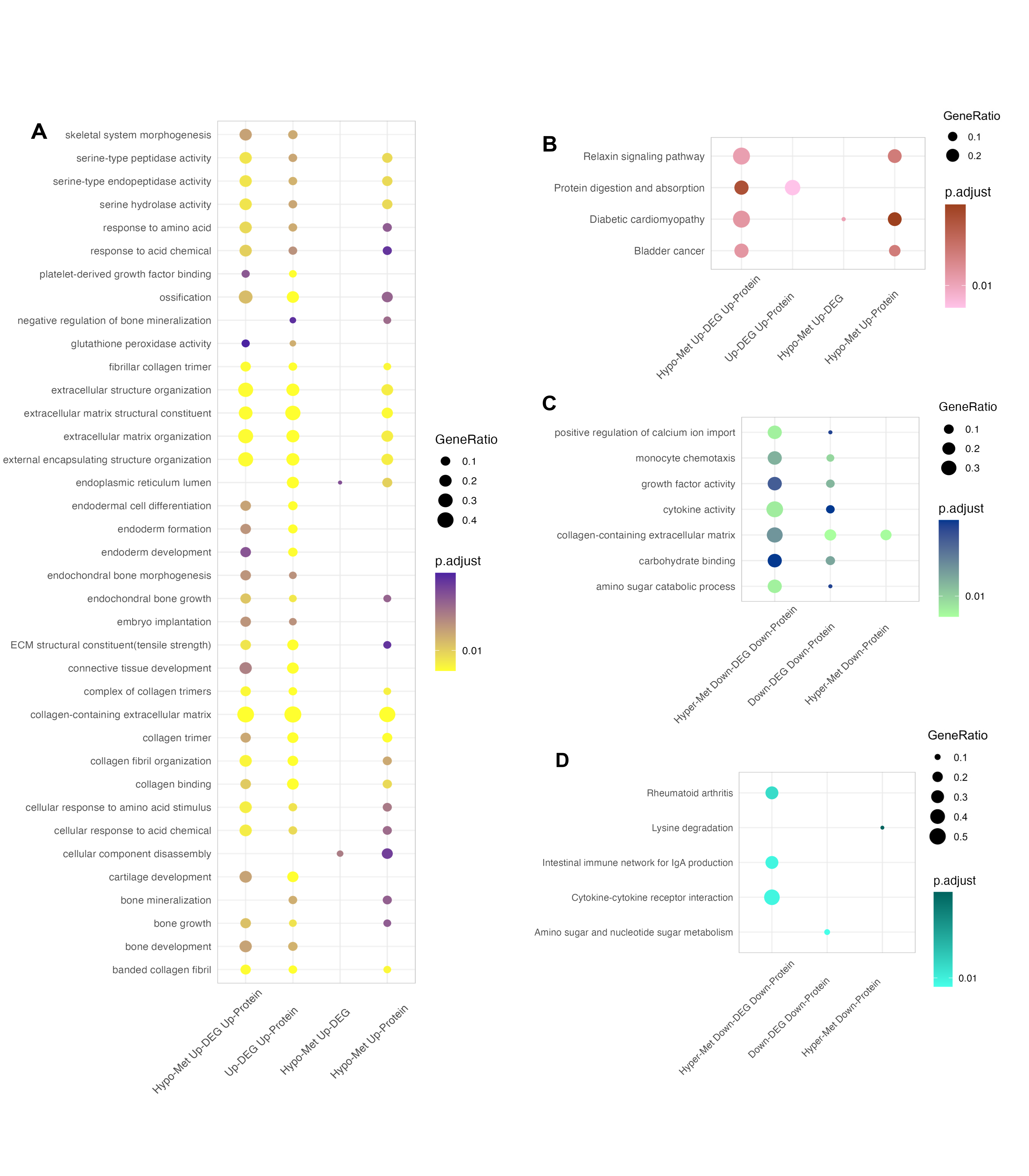

Supplement: Supplementary Figure 1 — GO and KEGG enrichment analyses at the two- or three-omics level. GO, Gene Ontology; KEGG, Kyoto Encyclopedia of Genes and Genomes. [file DataSheet1.zip › Supplementary Figure 2.tif]

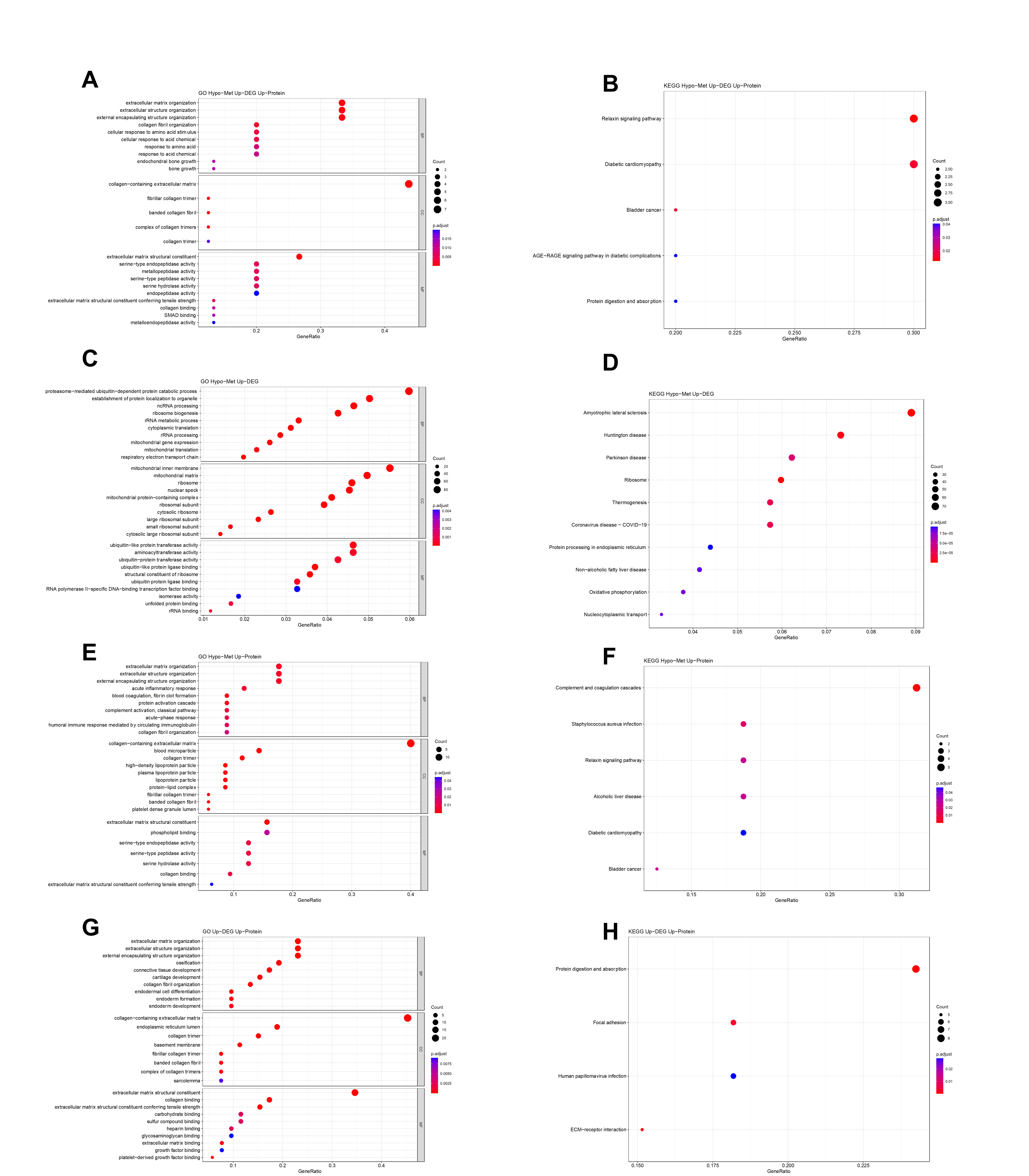

Supplement: Supplementary Figure 1 — GO and KEGG enrichment analyses at the two- or three-omics level. GO, Gene Ontology; KEGG, Kyoto Encyclopedia of Genes and Genomes. [file DataSheet1.zip › Supplementary Figure 3.TIF]

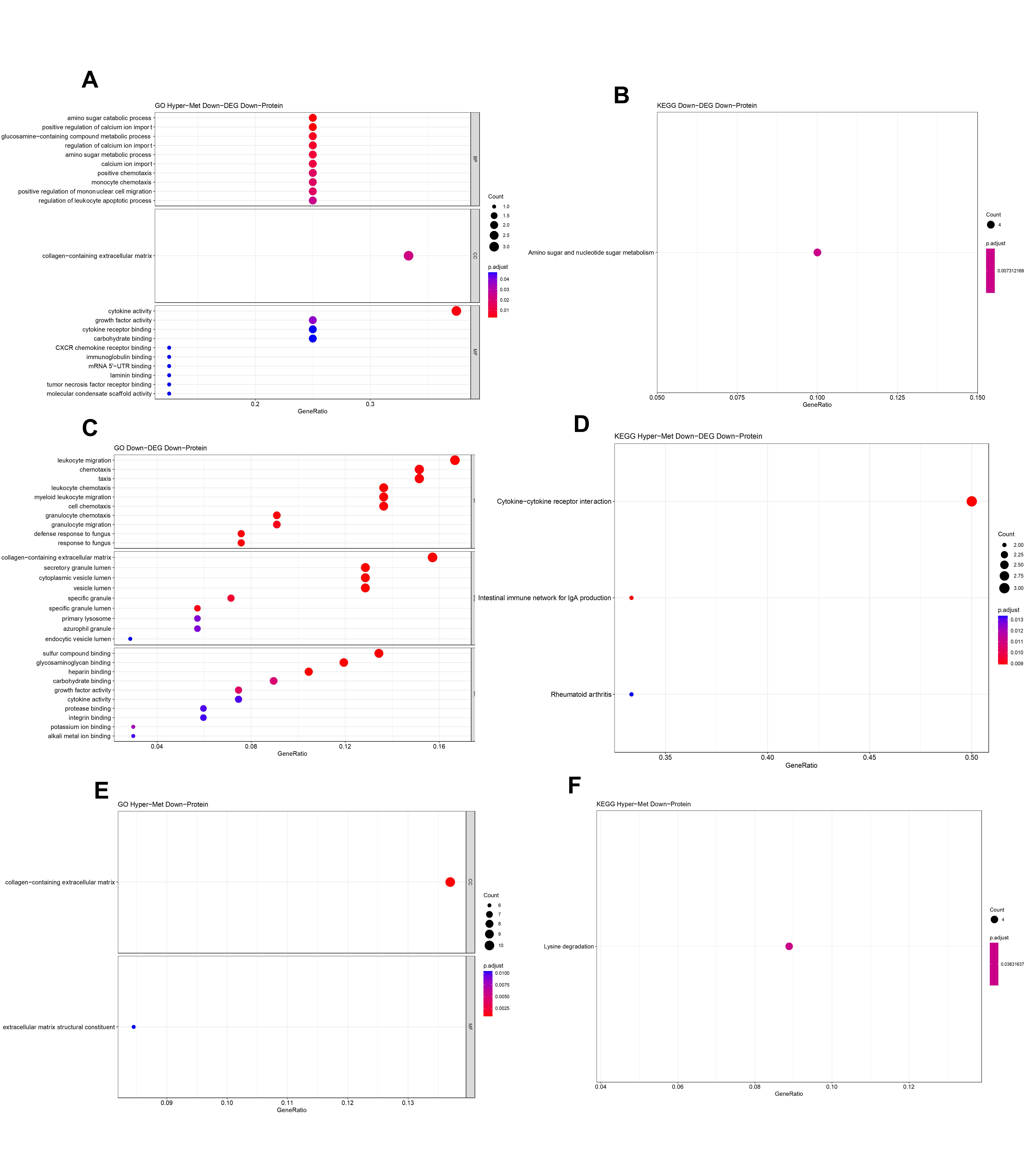

Supplement: Supplementary Figure 1 — GO and KEGG enrichment analyses at the two- or three-omics level. GO, Gene Ontology; KEGG, Kyoto Encyclopedia of Genes and Genomes. [file DataSheet1.zip › Supplementary Figure 4.TIF]
